# Kinetic and Equilibrium Studies of Doxorubicin Adsorption onto Carbon Nanotubes

**DOI:** 10.3390/ijms21218230

**Published:** 2020-11-03

**Authors:** Dorota Chudoba, Katarzyna Łudzik, Monika Jażdżewska, Sebastian Wołoszczuk

**Affiliations:** 1 Faculty of Physics, Adam Mickiewicz University, 61-614 Poznan, Poland; mojaz@amu.edu.pl (M.J.); sebastian.woloszczuk@amu.edu.pl (S.W.); 2 Frank Laboratory of Neutron Physics, Joint Institute for Nuclear Research, 141980 Dubna, Russia; katarzyna.ludzik@chemia.uni.lodz.pl; 3 Department of Physical Chemistry, University of Lodz, 91-403 Lodz, Poland

**Keywords:** adsorption, carbon nanotubes, drug delivery, doxorubicin, molecular dynamics simulation

## Abstract

This study provides deep insight into the adsorption process of doxorubicin onto different types of carbon nanotubes that have been proved to show attractive properties as a drug delivery system. The main aim of the work was to propose probable adsorption mechanisms and interactions between the anticancer drug and surface of modified and pristine carbon nanotubes at blood pH. The carbon nanotubes were oxidized to optimize the absorbance efficiency relative to that of pristine multiwalled carbon nanotubes. The adsorption isotherm of the modified system was well described by the Temkin equation. It confirms that the adsorption in the system studied involves also hydrogen and covalent bonding and is exothermic in nature. The experimental kinetic curves of adsorption were fitted to different mathematical models to check if the kinetics of doxorubicin adsorption onto the modified multiwalled carbon nanotubes follows a pseudo-second-order model and the chemical sorption is bound to be the rate-limiting. On the basis of the molecular dynamics simulation, it was shown that *in vacuo* the aggregation tendency of doxorubicin molecules is far more favorable than their adsorption on pristine carbon nanotubes (CNTs). It suggests that only functionalization of the nanotube surface can affect the interaction between doxorubicin and functional groups of the carriers and increases the efficiency of the drug loading process.

## 1. Introduction

Doxorubicin (DOX) is a structurally related cytotoxic antineoplastic antibiotic. As an effective anti-neoplastic agent, it is recommended for use in the therapy against several cancers such as leukemia, sarcoma, and many other solid organ tumors [1,2,3]. Nevertheless, DOX, like many cytotoxic drugs, has the disadvantage of non-specificity, which means that normal cells are destroyed as well as the cancerous tissues. Other numerous side effects of DOX toxicity, such as leukopenia and cardiotoxicity, limit the applicability of this drug [3,4,5,6,7]. For this reason, there is a justified need for the search for drug carriers that, without changing or weakening the mechanisms of drug action, would effectively and selectively transport the drug molecules to areas requiring treatment with minimum side effects. Over the past few decades, scientists have proposed and characterized many potential molecular-transport systems using microorganisms [8,9,10], liposomes [11,12], polymers [13,14], dendrimers [15], cyclodextrins [16], and biomolecular motors [17,18,19,20,21]. Despite several advantages of these proposed systems, most of them have so far proved to be insufficiently developed to be used in living organisms because of their toxicity, in vivo instability, or biochemical reactivity as well as issues with target-specific delivery or release [22]. Recently, attention has been focused on carbon nanomaterials, especially carbon nanotubes (CNTs), that provide many adaptation and application possibilities. Modulating the physicochemical properties of nanotubes through their functionalization is a key aspect of the potential applications of these exceptional materials in many fields [23,24,25,26]. Particularly high interest in modified carbon nanomaterials is associated with biomedical applications of CNTs [27,28,29] that are possible thanks to their chemical stability, bio-functionality, and biological compatibility. It is worth noting that CNTs have been successfully used for faster absorption of an antifungal antibiotic—amphotericin B [30]. Multiwalled carbon nanotubes (MWCNTs) modified with diaminotriethylene glycol have enabled the incorporation of antibiotic and fluorescein isocyanate, and the obtained systems do not exhibit negative effects on the human cells [31,32]. The undeniable advantage of CNTs, besides previously mentioned drug-targeting ability, are their own therapeutic anticancer properties after exposition to an infrared light source because they tend to generate heat up to 70–160 °C in a few seconds and thus produce hyperthermia [33]. In 2007, Gannonand and co-authors presented surprisingly optimistic results of experiments on living organisms that confirmed the effectiveness and selectivity of using CNT in the new photodynamic therapy [34]. An important argument indicating the distinctive application prospects of carbon nanomaterials is advanced studies of molecular complexes of folic acid photosensitizer from the porphyrin group and CNTs [35]. It has been demonstrated that the release of particles from carbon nanotubes can be stimulated by pH conditions [36,37]. This correlation opens great possibilities of using CNTs as cancer drug carriers as the tumor microenvironment is more acidic than the normal cells [38]. Therefore, there is much hope related to the possibility of using modified multiwalled carbon nanotubes as a platform or system for transporting many therapeutic or biologically active compounds such as drugs, DNA, and proteins into living organisms. Considering the needs of drug carrier design, the properties of carbon nanotubes, and the possibilities of their surface modification, it seems logical that they constitute a very attractive and important object of research. 

Many scientists have focused on the strategies of MWCNTs modification in order to increase their affinity to a given drug as well as optimize the operating conditions for the scale-up of the adsorption process [39].

The introduction of oxygen-containing groups such as carboxylic groups or amine groups on the surface of carbon tubes not only improves their dispersion and stability but allows attachment of different molecules, including drugs and proteins, through the formation of covalent bonds [40,41]. 

Shannahan has reported that functionalization of MWCNTs using microwave-assisted acid treatment increases the strength of binding proteins in comparison with that of pristine material [42]. Wang and co-authors have presented [43] that oxidation of MWCNTs induced by 8 h contact with H_2_SO_4_/HNO_3_ mixture (*v:v* = 3:1) at 30 °C enhances the adsorption capacity of DOX. Impressively efficient adsorption result was obtained after 10 days of incubation, but the reduction of contact time to 2 h caused a significant decrease in this parameter. It is worth emphasizing that an increase in the time of incubation causes a decrease in the release rate and loss of DOX bioactivity. Farahani has reported that on the surface of MWCNTs modified with HCl, the process of adsorption is easy and maximum adsorption was archived after 7 min. On the other hand, the efficiency of the process was poor [44].

Therefore, the choice of conditions of the functionalization process determines the degree of functionalization, the type of surface modification, but also destructions that play an important role in the adsorption process.

This paper reports a comprehensive study of adsorption of DOX on oxidized MWCNTs. To better understand the underlying mechanisms of DOX co-loading on carbon nanotubes, the interaction between the adsorbate and adsorbent in the method of molecular dynamics simulations was applied. A vital novelty of the study was a complementary application of experimental methods and computer simulations, which permits a better understanding of the underlying mechanisms of co-loading doxorubicin onto carbon nanotubes and interaction between the adsorbate and adsorbent.

## 2. Results and Discussion

### 2.1. Qualitative and Quantitative Determination of Surface Functional Groups on Modified Multiwall Carbon Nanotubes

#### 2.1.1. Zeta Potential

**Figure 1:**
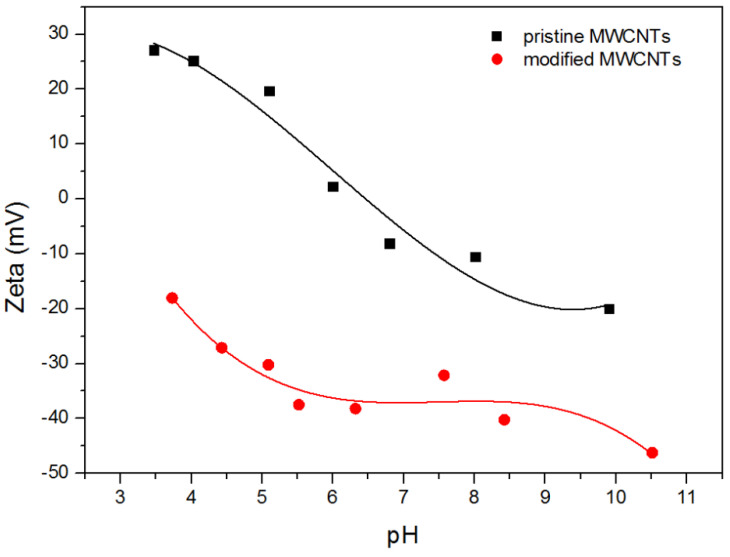
Zeta potential of pristine (**■**) and modified (**●**) MWCNTs vs. pH.

Zeta potential, a measure of electric charge of a dispersed particle, correlated with the oxidized species on the MWCNTs surface and their impact on the suspension behavior. The values of zeta potential as a function of pH for aqueous suspension of pristine MWCNTs and carboxylate MWCNTs are shown in Figure 1.

The results show that electrokinetic potential for unmodified MWCNTs decreases as the solution’s alkalinity increases, which changes after the isoelectric point. This tendency is due to the presence of some hydroxyl and carboxyl groups on the surface of pristine CNTs and probably due to the presence of amino-groups being residues of the process of synthesis. At acidic pH, amino groups and hydroxyl groups become protonated and, as a result, the surface of unmodified nanomaterials exhibits positive charge. Similarly, basic pH causes the gradual rise of dissociation degree of carboxylic groups and thus leads to an increase in the negative charge density of MWCNTs. The modified MWCNTs exhibit negative ζ potential over the whole investigated pH range. The decreasing tendency ζ = f(pH) can be attributed to the presence of numerous dissociated carboxylic groups and the suppression of the diffuse double layer [45,46]. A similar trend of ζ = f(pH), values of zeta potential, and isoelectric point for pristine MWCNT in water solutions have been reported by Sofía Gómez [39]. A bit lower values of the electrokinetic potential visible at acidic pH and its more negative values at higher pH range for pristine MWCNTs have been observed in KCl solution by Geng [47] and Pu [48]. Differences in the values of electrokinetic potential are inevitable and indicate that pristine material has different numbers of functional groups that determine the value of zeta potential. Analysis of ζ for carboxylated MWCNTs seems to be more complicated because the efficiency of oxidation strictly depends on the oxidizing agent, contact time with oxidant, as well as the temperature of oxygenation. Despite this, the values of electrokinetic potential for different modifications remain in good agreement with the results presented by us in References [39,49].

#### 2.1.2. Energy-Dispersive X-ray Spectroscopy

The qualitative and quantitative composition of the MWCNTs surface was established by EDS measurements. The results are presented in Table 1.

Elemental analysis of pristine MWCNTs provided the contents of nitrogen and oxygen that can be attributed to imino or amino groups; they can also come from hydroxyl or carboxyl groups. The detection of nitrogen is the evidence for the presence of imine or amine groups, while the detection of oxygen confirms the presence of hydroxyl or carboxyl groups. As can be seen, the chemical modification caused an increase in the content of oxygen after treatment with nitric acid from 0.94% to 2.79% that confirms the increase in the number of –COOH and -OH groups. The adsorption of DOX on MWCNTs led to the increased content of nitrogen, oxygen, and chlorine because of their presence in this drug. Furthermore, a marginal amount of sodium (1.42 wt %) was detected, which came probably from the PBS buffer.

#### 2.1.3. Thermogravimetric Analysis and Differential Scanning Calorimetry

**Figure 2:**
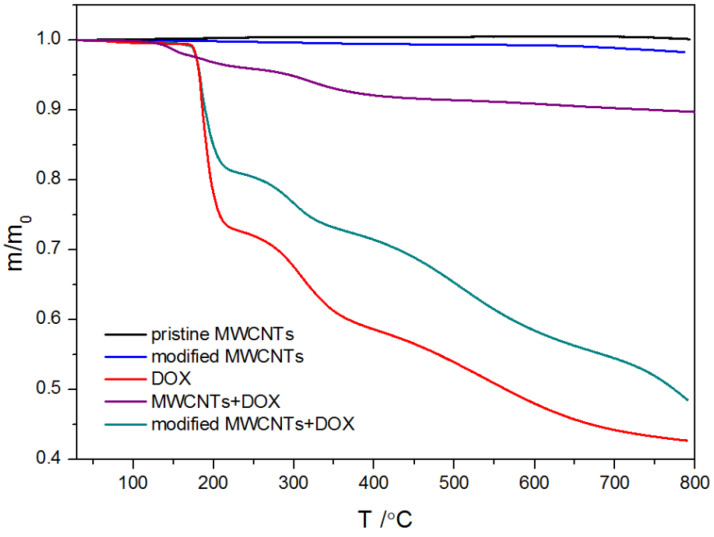
TGA curves determined under non-oxidizing atmosphere.

**Figure 3:**
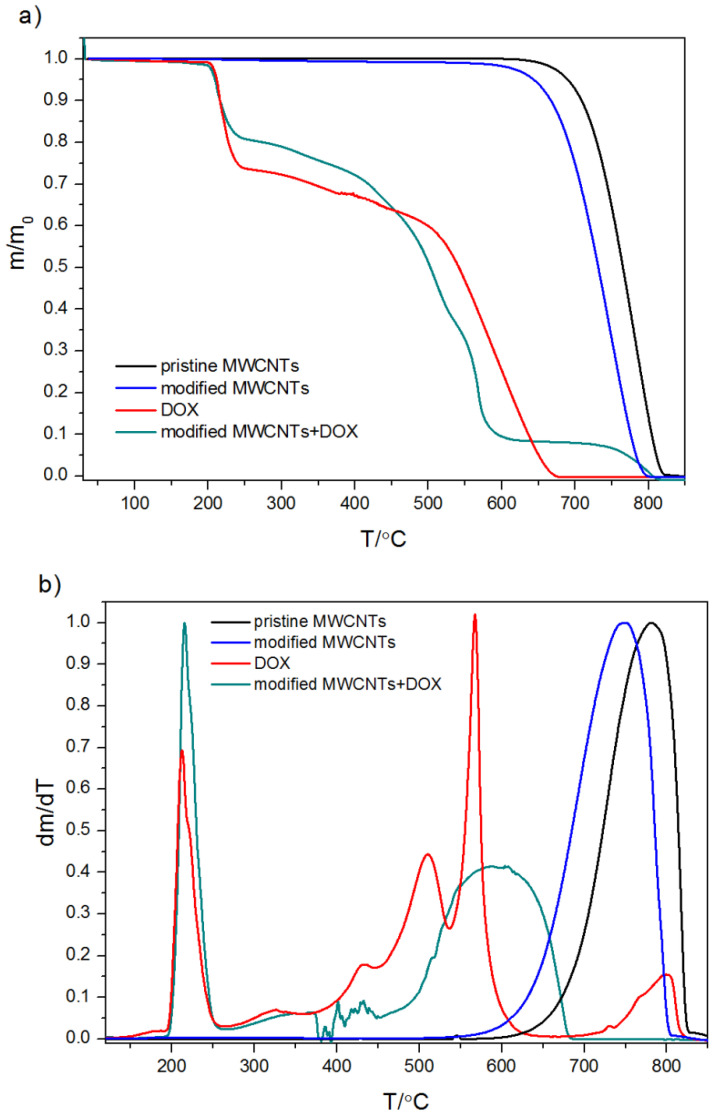
TGA curves determined under oxidizing atmosphere (**a**) and their first derivative (**b**).

TGA is a useful tool widely recommended in the characterization of nanomaterials not only in the investigation of processes such as adsorption but also to quantify the degree of functionalization, decomposition, and possible structural damage. For this reason, thermal analysis was carried out in an inert atmosphere or in an oxidizing atmosphere. The determined TGA curves are plotted in Figure 2 and Figure 3.

**Figure 4:**
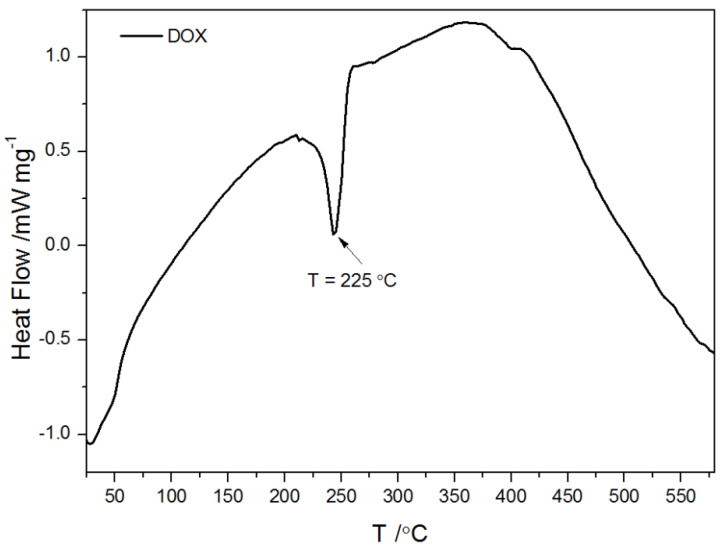
Differential scanning calorimetry curve of pure DOX.

The shape of TGA curves for pristine MWCNs and modified MWCNTs is typical. The minor weight loss (less than 0.5%) observed for all measured samples below 150 °C is interpreted as a result of the evaporation of the physisorbed water or gases. The descending tendency of the TGA thermal curve observed in the temperature range 120–500 °C is probably due to the decomposition of carboxyl and hydroxyl groups. Therefore, the mass loss within this temperature range is observed for acid-treated MWCNTs with groups generated during functionalization (Figure 2 and Figure 3) and it reflects the degree of functionalization. However, Zeta potential measurements and EDS results (Figure 1 and Table 1) indicated the presence of functional groups on the pristine CNTs. That is why it can be assumed that the number of −COOH and −OH groups in the unmodified CNTs is marginal as the mass loss is almost undetectable. The rapid mass loss in the range of 700–800 °C reflects mainly the thermooxidation of MWCNs. As follows from the curves, the onset of significant mass change is initiated at different temperatures for the modified and pristine MWCNTs. The shift of mass loss toward lower temperatures for acid-treated MWCNTs confirms that the chemical modification weakened the thermal stability of MWCNTs, probably from the oxidative effects of nitric acid used. The TGA curves obtained for pure DOX under oxidizing and non-oxidizing atmosphere showed a mass loss within temperature range 240–680 °C and 240–800 °C, respectively. The first well visible mass loss (27.5%), which starts in both cases at ~224 °C, can be attributed to defragmentation and melting, which were confirmed by differential scanning calorimetry (DSC) calorimetry (Figure 4). The TGA curves of CNTs modified with DOX prove that DOX is adsorbed on their surface area and in comparison, with the system CNTs-DOX, in the modified one, the amount of the drug is much greater (Figure 3).

The second jumpwise change in the curve is related to the degradation of the remaining amount of the drug. In the oxidizing atmosphere, the process ends at 680 °C when the mass of the samples reaches 0, in contrast to the decomposition under the nitrogen atmosphere, which is incomplete. The shift of the second stepwise mass loss on the curve of CNTs-DOX to lower temperatures results from greater susceptibility to oxidation of DOX deposited on the surface of CNTs, compared to that of the pure drug. Of note, the course of the second decrease of the TGA curve for MWCNT-DOX is not as smooth as in the case of pure DOX. It can be interpreted as a result of the development of covalent interactions between some DOX molecules and the modified MWCNTs. 

### 2.2. Adsorption of DOX on Carbon Nanotubes

#### 2.2.1. Computer Simulation for Adsorption of DOX on CNTs

**Figure 5:**
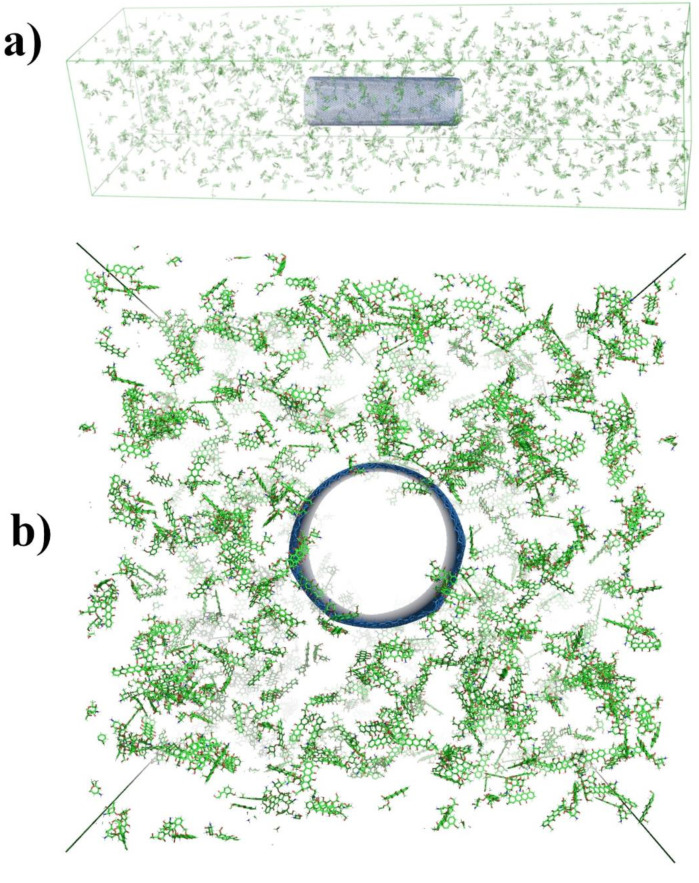
Snapshots of the input configuration from two points of view: (**a**) three-dimensional perspective; (**b**) view along the y-direction—we can see that initially the carbon nanotube was empty.

In order to observe the process of adsorption of DOX molecules inside the nanotube, we must make sure that in the input configuration there are no DOX molecules in the cylindrical volume with the diameter equal to that of the nanotube and running along the nanotube through the entire box (the y direction) (Figure 5b). The input configuration is equilibrated until the maximum force in the system reaches a value below 1 kJ mol^−1^ nm^−1^.

**Figure 6:**
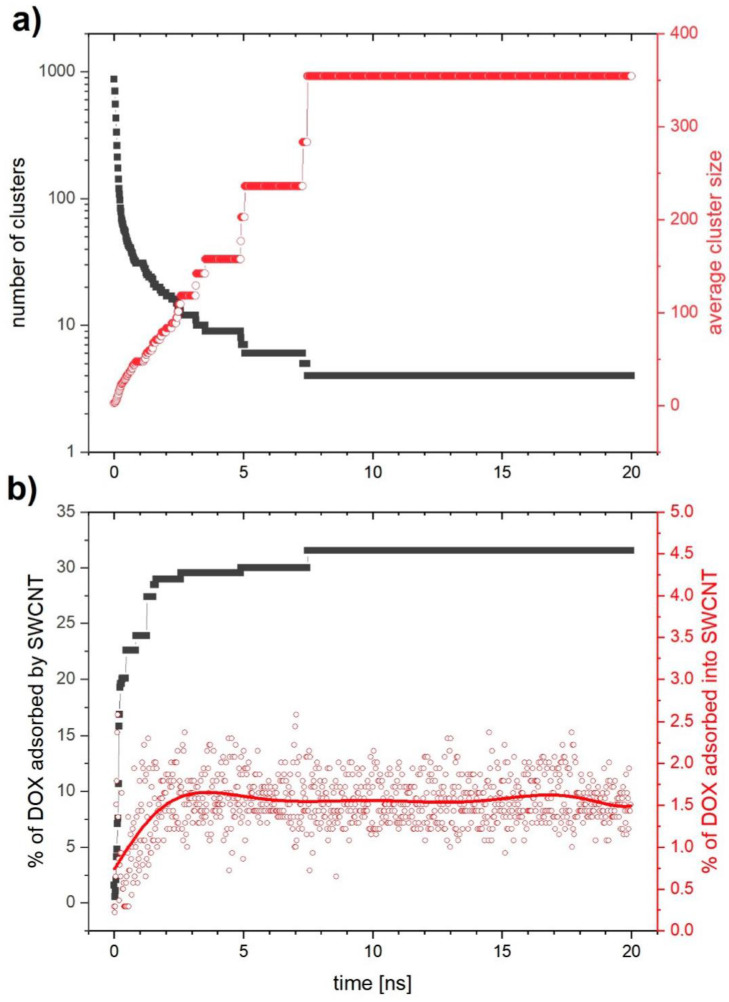
Molecular dynamics (MD) simulation of single-wall carbon nanotube: (**a**) the number of aggregates (gray) and mean degree of aggregation (red); (**b**) fraction of DOX molecules that are adsorbed onto CNTs (gray), and that are adsorbed inside the nanotube (red) as a function of time.

The simulation time was set to 20 ns, which is sufficient enough to track the process of aggregation of DOX molecules. First, we focus on the analysis of the simulation results obtained for an SWCNTs system. Figure 6a shows the number of aggregates (gray squares) and the average aggregate size (red squares) as a function of time. The number of aggregates decreases very fast from the values of the order of 1000 corresponding to small DOX aggregates, to the values below 10, characteristic for large aggregates. At the same time, the average aggregate size increases from a value of about 2.5 to a value close to 350 after about 7.4 ns and remains stable after that time. Figure 6b presents the percentage of all DOX molecules adsorbed onto the SWCNTs (gray squares) and the percentage of DOX molecules adsorbed inside SWCNTs (red squares). The fluctuations of the latter result from the algorithm of their counting. This algorithm divides the nanotube into single carbon rings arranged in parallel. For each of these rings, the algorithm calculates the number of DOX molecules whose center of mass lies inside the sphere of a radius smaller than that of the nanotube and coincides with the center of mass of the analyzed carbon ring. Then, from the sum of all rings, we eliminate duplicate DOX molecules. Each of the carbon rings of the nanotubes is deformed to some extent from the ideal circle in time, which causes fluctuations in the calculated number of DOX molecules that have been adsorbed into the nanotubes. According to Figure 6b, about 33% of DOX molecules are attached to SWCNTs covering them from the outside, while only about 1.5% are adsorbed on the inside. The small amount of DOX molecules that is adsorbed inside the nanotube is a result of two types of aggregation processes taking place simultaneously. One of them is the formation of aggregates exclusively by DOX molecules. The other involves the adsorption of both single DOX molecules as well as aggregated DOX clusters on the nanotube. Individual DOX molecules, due to their small size with respect to the diameter of the nanotube, can relatively easily enter the nanotube. The amount of free DOX molecules and small DOX aggregates drastically decreases over time and thus the probability of entering DOX into the nanotube drastically decreases. This explains the small number of DOX molecules inside the nanotube relative to their total adsorption on its surface.

**Figure 7:**
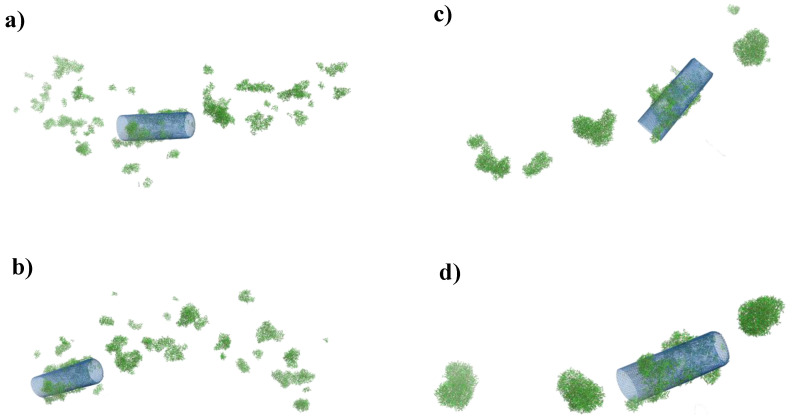
MD snapshots of a DOX (green) with a SWCNTs (blue), mass ratio 4(DOX):1(SWCNTs): (**a**) t = 0.5 ns; (**b**) t = 1 ns; (**c**) t = 5 ns; (**d**) t = 20 ns.

In Figure 7, we present the visualization from MD simulation for four simulation time values: (a) 0.5 ns, (b) 1 ns, (c) 5 ns, and (d) 20 ns. We can see that over time, clusters of freely aggregating DOX molecules merge into larger structures with both DOX and DOX-SWCNTs aggregates. Finally, after the time about t = 7.5 ns, we observe four aggregates, one of which is DOX-SWCNTs, while the other three are made of pure DOX (Figure 7d).

**Figure 8:**
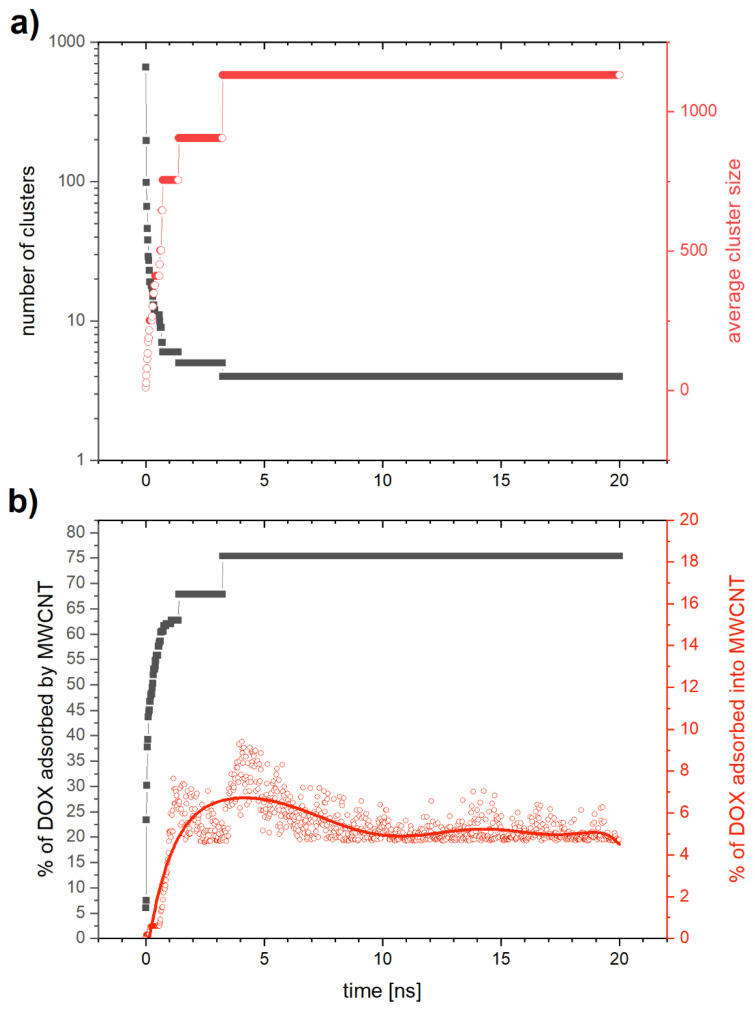
MD-simulated MWCNTs with DOX: (**a**) the number of clusters (gray) and mean degree of aggregation of single aggregate (red) as a function of time; (**b**) fraction of DOX molecules adsorbed on the multiwall carbon nanotube (gray), and that adsorbed inside the multiwall carbon nanotube (red) as a function of time.

Figure 8a presents the results for MWCNTs with DOX as a function of time. Gray squares represent the number of aggregates and red squares the average aggregate size. We can see that the aggregation process is similar to that observed for SWCNTs. The number of aggregates decreases fast reaching the value of (=4) after about 3 ns, while the average cluster size increases rapidly. This process is about twice as fast as in the case of SWCNTs and we also notice two parallel processes of aggregation. One of them is the aggregation of DOX molecules into free clusters that merge with time increasing their size, while the second one is the aggregation of DOX onto and into the MWCNTs. Figure 8b displays the time evolution of the percentage fraction of DOX molecules that form aggregates with MWCNTs (gray squares), and the part of them that aggregated inside MWCNTs (red squares). We see that after 3 ns aggregation is saturated with about 75% of the DOX molecules bound to the MWCNTs, of which about 5% are aggregated inside the nanotube. This value is more than twice as high as in the case of SWCNTs.

It is worth emphasizing that in the considered systems the tendency to form molecular aggregates between drugs molecules can be treated as a competitive effect that weakens the effectiveness of the adsorption process. What is more, in an aqueous environment, the solvent effects (hydrophobic hydration and solvation) are known to obstruct adsorption. Therefore, it can be assumed that the adsorption on the hydrophobic, unmodified carbon nanotube will be less efficient than obtained in the presented simulation.

#### 2.2.2. Adsorption Isotherm of DOX Adsorption on Pristine MWCNTs and Modified MWCNTs

**Figure 9:**
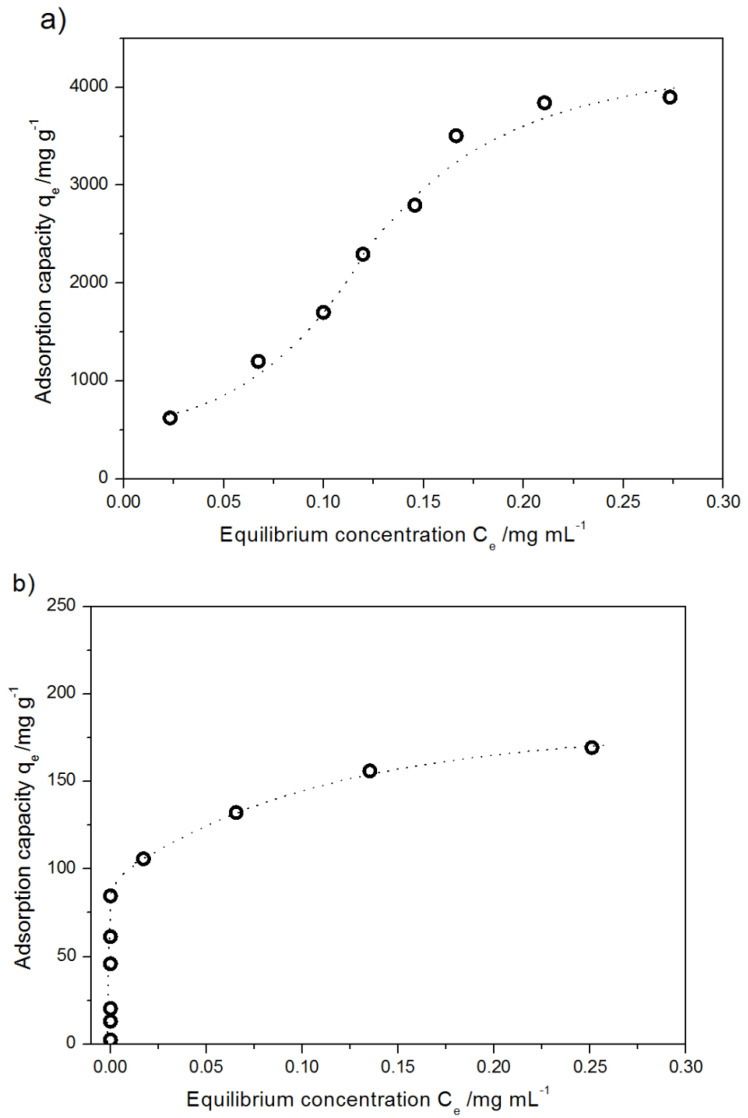
The average adsorption isotherms of DOX at 298.15 K on (**a**) modified MWCNTs (**b**) pristine MWCNTs. Data are presented as mean ± SD (*n* = 3).

The isotherm was recorded at 298.15 K for different initial concentrations of DOX, from 0.067 mg mL^−1^ to 0.533 mg mL^−1^ and from 0.00016 mg mL^−1^ to 0.364 mg mL^−1^ for the modified and pristine MWCNTs, respectively (Figure 9).

The maximum adsorption capacity of acid-treated MWCNTs was ca 3800 mg g^−1^, while that of pristine MWCNTs was ca 175 mg g^−1^. The adsorption isotherms reach a plateau at *q_e_* of 3800 mg g^−1^ and 150 mg g^−1^ for the modified and unmodified systems, respectively. The initial concentrations for which the isotherms were recorded were 0.53 mg mL^−1^ and 0.3 mg mL^−1^ for the oxidized and unmodified systems, respectively. Interpretation of the results was performed assuming three models of isotherms.

In the Freundlich model, the empirical equation is based on adsorption on a heterogeneous surface or microporous surfaces. As a result, the stronger binding sites are occupied at first and the binding tendency decreases with the increasing degree of site occupation. The Freundlich isotherm is expressed by Equation (1) [50]:

(1) qe=kFCe1n

where *q_e_* (mg g^−1^) is the amount adsorbed of DOX per unit weight of adsorbent at equilibrium, $k_{F}$ represents a constant related to adsorption capacity (mg g^−1^); 1/n is an empirical parameter describing adsorption intensity. The Freundlich isotherm coefficients obtained from the linear plots of *lnqe* vs. *lnCe* for modified and pristine carbon nanotubes are collected in Table 2. 

The value of parameter *1/n* (0.1 < *1/n* < 1) indicates a favorable adsorption of DOX in experimental conditions on the modified MWCNTs [51]. Another model considered that was proposed by Langmuir, which is commonly used for the description of monolayer adsorption on a finite number of identical sites [52]. The Langmuir isotherm is expressed as:

(2) $$\frac{C_{e}}{q_{e}}=\frac{C_{e}}{q_{max}}+\frac{1}{q_{max}K_{L}}$$

where $q_{max}$ is the maximum adsorption capacity and $K_{L}$ (mL g^−1^) is the Langmuir constant related to the affinity of the binding sites and energy of adsorption.

The parameters of the Langmuir equation obtained for pristine MWCNTs are presented in Table 2. The Langmuir’s maximum adsorption capacity *q_max_*= 185.2 ± 20.4 mg g^−1^ well corresponds to the experimental value of ca 175 mg g^−1^. The fit of the results for the modified MWCNTs to the Langmuir isotherm was unsatisfactory, so this model was no longer taken into account. This simple model is based on the assumption that the process of adsorption takes place on a homogenous surface endowed with equivalent adsorption sites, with neither bond generation nor interactions between the adsorbed molecules. That is why the poor fit of the model to the results seems logical. The correlation coefficient calculated assuming the Freundlich model (R^2^ = 0.9658) confirms the heterogeneous distribution of active sites and well represents the nature of the process. On the basis of the tight fit of Langmuir isotherm, many authors have suggested single-layer adsorption of DOX on CNTs [53,54]. Despite good correlation factors obtained assuming the Langmuir model to adsorption process of DOX onto modified MWCNTs, Wang and co-authors have proved by the morphological observation that in the above-mentioned case the adsorption is not of single-layer type [43]. Our experimental results confirm that the formation of a monolayer by DOX molecules on the modified MWCNTs is hardly possible. It is worth noticing that the presented simulation of DOX adsorption on CNTs also suggests that their coverage by a monolayer is implausible (Figure 7). A similar conclusion has been obtained for MWNTs modified with polyethylene glycol [44,55].

For the above-mentioned reasons, the Temkin isotherm model was applied. This model describes the effects connected with indirect adsorbate/adsorbate interactions during the adsorption process and permits determination of the Temkin constant *b* that is related to the heat of sorption [56,57]. The linear version of Temkin isotherm is expressed as:

(3) $$q_{e}=\frac{RT}{b}lnk_{T}+\frac{\mathrm{RT}}{b}lnC_{e}$$

where *b* is Temkin constant which is related to the heat of sorption kJ mol^−1^ (*ΔH = −b*) and *k_T_* is Temkin isotherm constant (mL g^−1^).

The values of coefficients obtained assuming the Temkin model of adsorption collected in Table 2 may suggest that the process of DOX adsorption on carbon nanotubes can be treated as chemisorption. Moreover, the process is exothermal in nature for both types of nanotubes. It is worth noticing that the heat of sorption differs diametrically for oxidized and pristine systems that suggest competitive phenomena, from the point of view of energy effects and different ways of adsorption. Considering adsorption processes for different systems with MWCNTs, it can be concluded that the vast majority of other authors report exhibit low (near zero) but a positive value of the heat of sorption [58]. Temkin equation allows a description of the adsorption process for modified carbon nanotubes (Langmuir equation). It confirms different mechanisms of DOX adsorption in the mentioned cases. The non-ideal fit of the Temkin equation to the experimental data can be a consequence of a great range of ion concentration [59]. For this reason, the low value of Temkin constant k_t_, which can suggest a weak interaction between the DOX and the sorbent, should be treated with caution and should not lead to a further strict conclusion [60].

#### 2.2.3. Kinetics of Adsorption

In order to optimize the adsorption conditions, the efficiency of adsorption as well as conditions for the scale-up of the kinetic aspects of the process seem to be crucial.

**Figure 10:**
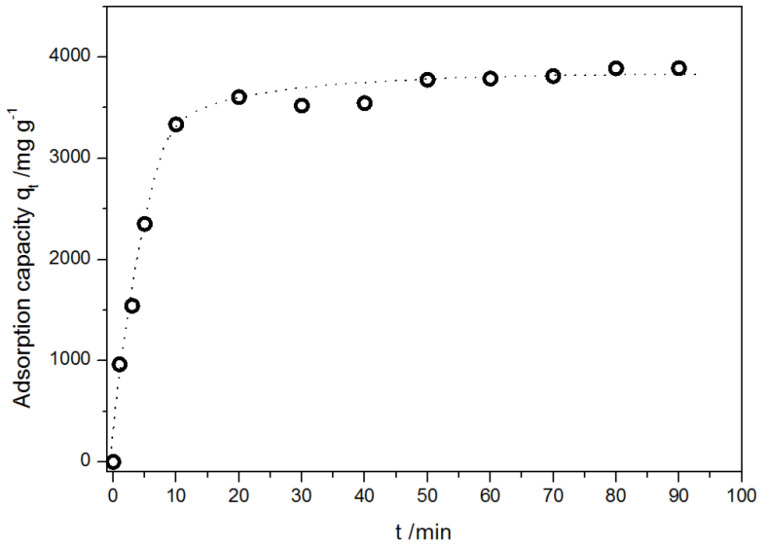
Kinetics of DOX adsorption on the modified MWCNTs at 298 K. Data are presented as mean ± SD (*n* = 3).

The experimentally obtained adsorption kinetic curve of DOX onto modified MWCNTs is presented in Figure 10.

The adsorption process was very rapid; for the first 10 min, the adsorption capacity reached 3335 mg g^−1^. It may be attributed to contacts of drug molecules with available surface adsorption sites on the outermost layer of MWCNTs, while subsequent gradual adsorption may be attributed to uptake or incorporation of the drug molecules into the adsorbents as shown in MD calculations. The equilibrium was reached after 15–20 min. 

In order to characterize the adsorption process of DOX on the modified MWCNTs and potential rate-controlling steps, various kinetics models, namely, pseudo-first order, pseudo-second order, Elovich equation, intra-particle diffusion model, and fractional power kinetic model were applied [50,61,62]. The original equation of the used models is:

(4) $$\log\left(q_{e}-q_{t}\right)=\log q_{e}-\frac{k_{1}}{2.303}t\mathrm{pseudo}-\mathrm{first}\;\mathrm{order}\;-\;\mathrm{Lagergren}\;\mathrm{equation},$$

where $k_{1}$ is the Lagergen rate constant of adsorption (min^−1^) and $q_{e}$ and $q_{t}$ are the adsorption capacity (mg g^−1^) and the adsorption capacity at time t:

(5) $$\frac{t}{q_{t}}=\frac{1}{k_{2}q_{e}^{2}}+\frac{t}{q_{e}}\mathrm{pseudo}-\mathrm{second}\;\mathrm{order}\;-\;\mathrm{Ho}-\mathrm{McKay}\;\mathrm{equation},$$

where $k_{2}$ is a rate constant of adsorption/mg g^−1^ min^−1^. The adsorption capacity at time t following the intra-particle diffusion model, Weber and Morris, is calculated as:

(6) $$q_{t}=k_{i}t^{\frac{1}{2}}+C_{i}$$

where $k_{i}$ is the rate constant of intra-particle diffusion at stage i and $C_{i}$ reveals the thickness of the boundary layer of intra-particle diffusion model. The fractional power kinetic model is expressed as:

(7) $$ln\;q_{t}=lnK_{FP}+vlnt$$

where $K_{FP}$ is a constant, and *v* is also a constant that is usually lower than unity if adsorption kinetic data fit well into the power function model. 

The adsorption capacity at time t described by the Elovich model is:

(8) $$q_{t}=\frac{1}{\beta }ln\alpha \beta +\frac{1}{\beta }lnt$$

where *α* and *β* are the Elovich coefficients, *α*/mg g^−1^ min^−1^ is related to chemisorption rate, and *β*/g mg^−1^ is a constant that depicts the extent of surface coverage.

**Figure 11:**
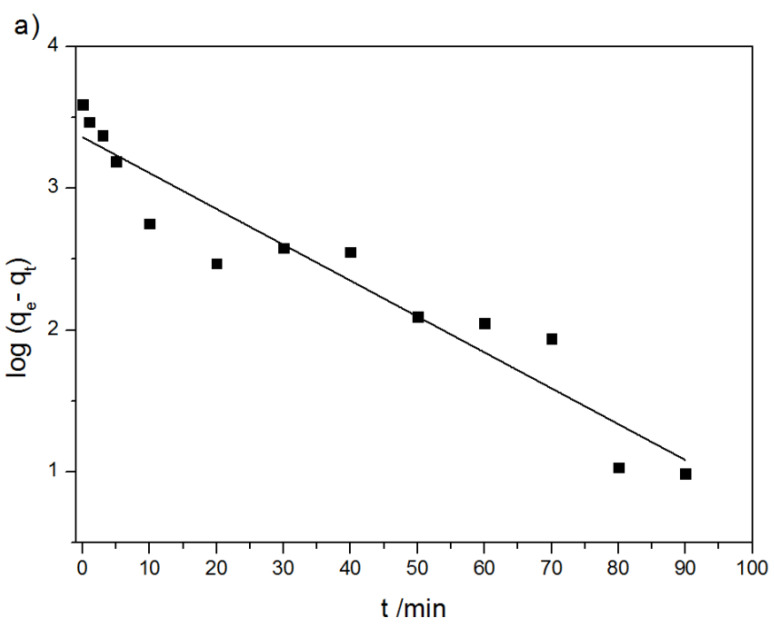

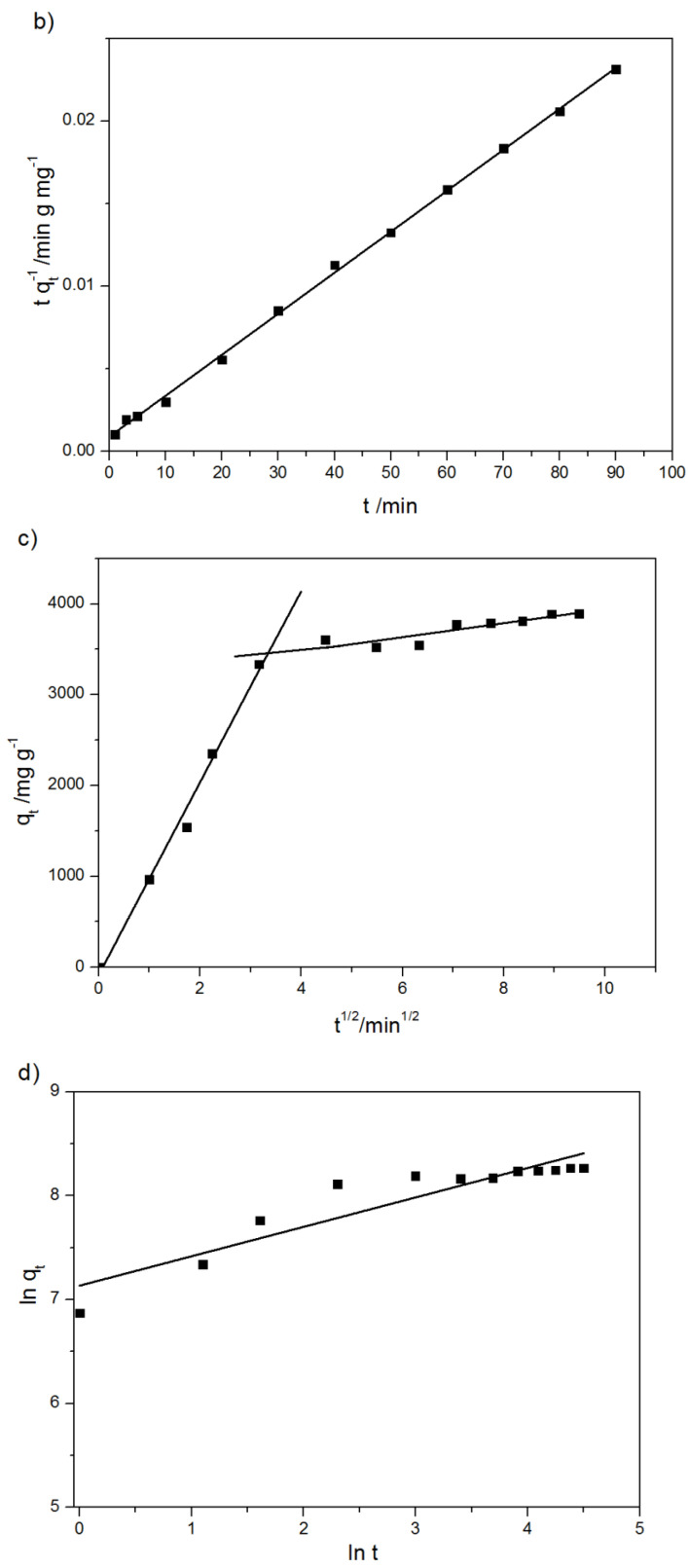

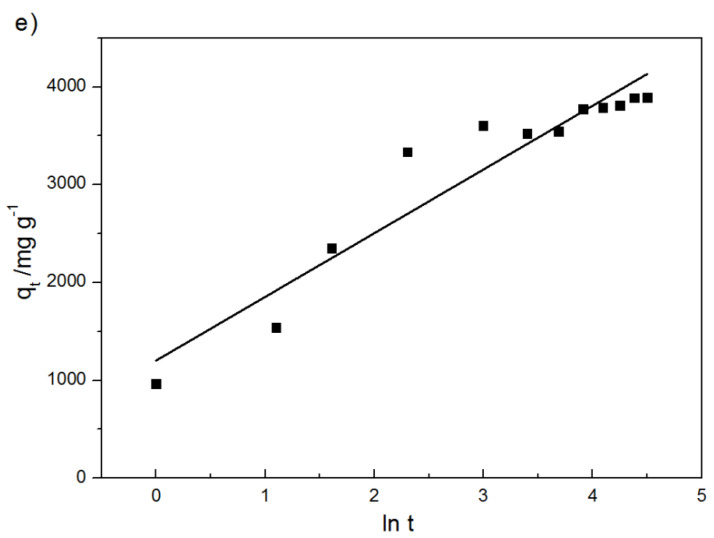
Pseudo-first order plot for the uptake of DOX on MWCNTs (**a**), pseudo-second order plot for the uptake of DOX on MWCNTs (**b**), intra-particle diffusion model plot for the uptake of DOX on MWCNTs (**c**), fractional power model for the uptake of DOX on MWCNTs (**d**), the Elovich plot for the uptake of DOX on MWCNTs (**e**). Data are presented as mean ± SD (*n* = 3).

The parameters of the above-mentioned kinetics models and the validation results are given in Table 3 and depicted in Figure 11a–e.

The best fit was obtained for the pseudo-first and pseudo-second order models. However, the value of equilibrium sorption capacity from the pseudo-first order model (q_e_ = 2290 mg g^−1^) was significantly higher than the experimental value of 3900 mg g^−1^. It indicates that this model is not appropriate for describing the investigated phenomena. Thus, the interpretation of parameter obtained from the model is useless. In contrast, the q_e_ parameter calculated from the pseudo-second order equation (q_e_ = 4029 mg g^−1^) matches the experimental value (3900 mg g^−1^) (Table 3). The values obtained assuming the pseudo-second order equation (R^2^ coefficient) provide a basis for deeper analysis of the DOX sorption mechanism. It can be said that the nature of the adsorption process can be potentially treated as chemically controlled to a large extent [63]. The presented suggestion is confirmed by the high value of the Elovich constant α that is related to chemisorption. In comparison with the literature data, the value of maximal q_e_ reported in this study is much higher than values obtained for SWCNTs (1700 mg g^−1^) [64] as well as for oxidized MWCNTs after 2 h of the adsorption process (q_e_ < 1000 mg g^−1^) [43]. Wang et al. have reported also that the increase of time contacts to 10 days for the system modified MWCNTs – DOX allowed an increase in the adsorption capacity till 8000 mg g^−1^ [43]. Nonetheless, the ultra-long adsorption equilibrium time affects the removal rate as a consequence of the formation of stable sludge-like structure and loss of bioactivity of the drug. 

The value of the second rate constant of the adsorption process (k_2_) 6.92 × 10^−5^ g mg^−1^ min^−1^ obtained assuming the model of pseudo-second order proves that the process of adsorption is much faster than of DOX adsorption on the carrier modified by Wang [43] (3.52 × 10^−8^ g mg^−1^ min^−1^), however, it is much slower than that of adsorption of lysozyme [65] or curcumin [55] on MWCNTs modified in different ways. 

The multilinear profile of the Weber–Morris plot that did not pass through the origin of the coordinate system indicates more than one diffusion step. The rapidly increasing first curve (k_1_ >> k_2_) Figure 11c suggests that intra-particle diffusion mainly controls the uptake of DOX on the modified MWCNTs, but what is more, is also affected by the boundary layer diffusion process [45,46]. As time passes and the DOX concentration in the solution drastically decreases, intramolecular diffusion is no longer as efficient as at the beginning of the process, which results in a weaker upward trend of the function f = f(t^1/2^) 11(c).

#### 2.2.4. Mechanism of Adsorption

On the basis of the data presented, it can be concluded that the drug adsorption capacity of the modified MWCNTs is high and the rate of adsorption is high and fast. The explanation of the phenomena is based on three factors: the unique structure, electrostatic interaction, and the ability to bond formation. Generally, the structure of MWCNTs permits the contact between the carbon atoms and the drug molecules and thus enables the interaction between them. However, the first layer of carbon atoms in MWCNTs is more exposed to contact with the drug molecules than the deeper ones. The molecules of DOX can also locate inside nanotubes but the probability and efficiency of this phenomenon are meaningless in comparison with adsorption on the MWCNTs. The structure of oxidized material with nanodefects (confirmed by TGA analysis) and functional groups (TGA and EDS) cannot be treated as a homogenous and potential adsorption sides as equivalent. The electrostatic interactions depend mainly on the charge of the surface of MWCNTs. As shown on the basis of zeta potential measurements, the modified MWCNTs exhibit a negative charge in the whole pH range considered. Thus, the positively charged molecule of DOX can be easily attracted and adsorbed onto the career. The charge on the surface of pristine MWCNTs strongly depends on the pH value (Figure 1). Although the surface of the nanotube is negatively charged at alkaline pH, the charge is much lower when compared to that on the modified carrier. Therefore, electrostatic interactions are less effective and the adsorption less efficient. The ability to form bonds affects the adsorption capacity of the material. It is associated with the presence of functional groups in both the DOX molecule (OH, COOH, NH_2_) and nanotubes (OH, COOH). The possibility of hydrogen-bonding between OH-COOH, NH_2_-COOH, OH-NH_2,_ and OH-OH groups increases the adsorption capacity of the modified CNTs. Moreover, modified MWCNTs can form also an ester bond between the primary hydroxyl group of the chemotherapeutic agent and the carboxyl group located on the surface of the nanocarrier [66].

Considering the driving forces and mechanisms of adsorption of DOX on the surface of nanotubes, it is worth emphasizing that non-oxidized MWCNTs act as nano-reservoirs adsorbing drug molecules by host–guest interaction. The hydrophobic and π–π stacking interaction force between the chains of adsorbed molecule and surface of CNTs play a key role in adsorption and do not affect the electronic network of the tubes [67,68]. In contrast, the adsorption process of the modified MWCNTs involves not only physisorption, electrostatic, and π–π stacking interactions but mainly hydrogen and covalent bonding. The kinetic studies suggest that the adsorption of the drug on the modified MWCNTs depends on the types of adsorbent and adsorbate and involves chemisorption as well as physisorption.

## 3. Experimental

### 3.1. Materials

MWCNs with average outer diameter of 9.5 nm and a length of 1.5 µm were obtained from Nanocyl SA (Sambreville, Belgium). They were produced via the catalytic chemical vapor deposition (CCVD) process and then purified up to 95 wt %. Doxorubicin hydrochloride (purity ≥ 98%, Sigma Aldrich) was used without further purification. All solutions were prepared in Milli-Q (The Millipore ultrapure water Co., Ltd., Millipore, Burlington, MA, USA) ultrapure water.

#### Functionalization of MWCNTs

**Figure 12:**
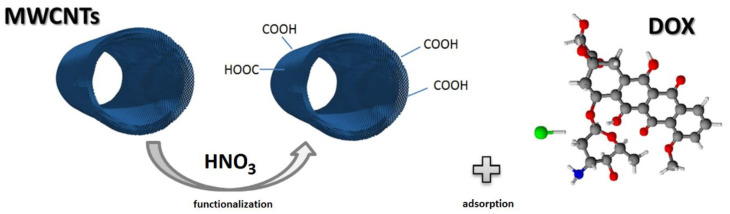
Schematic of multiwalled carbon nanotubes (MWCNTs) modification and doxorubicin (DOX) adsorption.

MWCNTs were subjected to exohedral functionalization by modifying the external walls of CNTs or their ends with oxidizing acids. As a consequence, it was possible to generate such functional groups as –COOH, –OH, or –COOCl at the sites of defects and at the ends of nanotubes. (Figure 12)

MWCNTs were modified by treatment in boiling 8 M nitric acid for 2 h. The ratio of MWCNTs to HNO_3_ (aq) (in g:dm^3^) was 0.75:0.1. The acid treatment leached the residual catalyst particles and introduced surface functional groups of acidic properties. The procedure of chemical modification of MWCNTs was repeated three times. The decrease in the mass of MWCNTs was 2.5 ± 0.2% in each case. The as-obtained product was rinsed several times with ultrapure deionized water (Merck, Darmstadt, Germany) until the pH of supernatant reached 7 and subsequently dried in air for 24 h at 120 °C.

### 3.2. Methods

#### 3.2.1. Thermal Analysis

The differential scanning calorimetry (DSC) thermogram of pure DOX was recorded using Netzsch DSC 204 F1 Phoenix in the temperature range 20–600 °C and heating rate of 5 deg/min. The thermogravimetric analysis experiments were carried out in non-oxidizing (100% nitrogen) or oxidizing (5% oxygen, 95% nitrogen) atmosphere at the heating rate of 10 deg/min using TA Instruments Q50. The temperature range of the experiments was 25–800°C for the nitrogen atmosphere and 25–900 °C for the oxidizing atmosphere.

#### 3.2.2. UV–VIS Spectroscopy

The concentrations of DOX were determined by means UV–Vis spectrometer Shimadzu 2401 (SHIMADZU Enterprise Management Co., Ltd., Shimadzu, Japan) at λmax = 484 nm [39].

#### 3.2.3. Zeta Potential

Electrokinetic potential values of MWCNTs and the modified MWCNTs were measured in deionized water in the 3–10 pH range by means of Litesizer™ 500 Anton Paarat at room temperature. Suspensions were prepared to get the particle concentration of 1 mg/mL. The pH value of the investigated systems was adjusted with HCl and NaOH. Before measurement, 30 min ultrasonic bath treatment (at 44 MHz) was applied. The values of zeta potential presented in this paper represent the mean average of triplicate measurements.

#### 3.2.4. Adsorption of DOX on Functionalized MWCNTs

The adsorption isotherms of DOX on modified MWCNTs at 298 K were determined using three liquid phases: (I) the aqueous solution of DOX 1 mg mL^−1^, (II) the suspension of modified MWCNTs in water 1 mg mL^−1^, and (III) PBS (phosphate-buffered saline, pH = 7.4). The experiment involved the preparation of mixtures of these liquid phases, with always the same volume of modified MWCNTs suspension of 300 μL. The volume of DOX aqueous solution varied between 0 (blank) and 2400 μL, and the volume of PBS varied between 4200 and 1800 μL. The total volume of these three phases was always of 4500 μL. The mixtures were shaken for 24 h. Then, the carbon nanotubes were separated from the suspension via centrifugation. The concentration of DOX was determined via the spectrophotometric standard curve method. The adsorbed amount of drug per unit weight of adsorbent-modified and nonmodified MWCNTs (mg g^−1^) at equilibrium was calculated according to Equation (1) [69]:

(9) $$q_{e}=V\frac{C_{0}-C_{e}}{m}$$

where *m* (g) is the mass of nanotubes, *V* (mL) is the total solution volume, *C*_0_ and *C_e_* mg mL^−1^ represent the initial and equilibrium concentrations of DOX, respectively. 

#### 3.2.5. Determination of Adsorption Kinetics

The kinetic curve of DOX adsorption on modified MWCNTs was determined as described above at 298 K. The initial concentration of DOX was chosen on the basis of the adsorption isotherm (the concentration at which the isotherm reached maximum). The obtained mixtures were shaken for 1, 3, 5, 10, 20, 30, 40, 50, 60, 70, 80, and 90 min, respectively. Next, the concentration of DOX was determined by the spectrophotometric standard curve method [69]. In order to evaluate the effect of contact time on the amounts of DOX adsorbed on modified and nonmodified MWCNTs, the adsorption capacity at time *t* was calculated according to Equation (2):

(10) $$q_{t}=V\frac{C_{0}-C_{t}}{m}$$

where *m* (g) is the mass of adsorbent, *V* (mL) is the total solution volume, and *C*_0_ and *C_t_* (mg mL^−1^) represent the initial and time (*t*) concentrations of DOX.

#### 3.2.6. Molecular Dynamics Simulations

Molecular dynamics (MD) simulations were performed using GROMACS [70] software package and OPLS-AA force field [71,72]. MD calculations were performed using the canonical (NVT) ensemble (constant number of particles, volume, and temperature) at 293.15 K using a v-rescale thermostat with the time constant for coupling (=0.1 ps). Electrostatic interactions were calculated using the pme-based algorithm, and the LINCS algorithm [73] was used to constrain all the bonds to the forcefield equilibrium lengths. The time step, *dt*, was set to the value (=1 fs), which ensured optimum performance on the one hand and made the system stable (permitted avoiding runway) on the other hand. The cutoff for the non-bonded interactions (electrostatic and Van der Waals) was set to 1.0 nm, which is the default value for OPLS-AA forcefield. The potential-shift modifier was chosen both for Van der Waals and electrostatic interactions. The simulation time was set to 20 ns, which allowed us to observe all the processes of interest and describe them quantitatively. The simulations were performed in vacuo. Limitations of computational performance in the case of computer simulations induced us to build relatively small models when compared to systems on a real scale. Real systems usually show differentiation of certain important parameters, which in the computer model must be set precisely because of the relatively small scale. In the experimental system, both the density of nanotubes and the density of DOX at the beginning of the experiment were not constant and could vary significantly depending on a certain small volume comparable to the computer model. In the computer model, however, we need to fix the initial average density of both the nanotubes and the DOX. The simulation box was set to 20 × 80 × 20 nm. In order to check the dependence of the efficiency of adsorption with respect to the type of nanotube, we simulated the DOX with a single-wall zigzag nanotube (SWCNT) or with a three-wall zigzag nanotube (MWCNT). Nanotubes were generated using the buildCstruct script [74] adapted by us to our needs. In the case of MWCNT, three SWCNTs with specific diameters were generated and arranged coaxially into one MWCNT. The diameter of the nanotubes was chosen to match the diameter of the nanotubes in the experimental system. In the case of SWCNT, it was ϕ = 6.42 nm, while in the case of MWCNTs it was ϕ_1_ = 6.42 nm, ϕ_2_ = 7.12 nm, and ϕ_3_ = 7.83 nm, respectively. The length of the nanotube was set to L = 20 nm to ensure a reasonable simulation time on the one hand, and not to affect the observed phenomena by too short nanotube on the other hand. The number of DOX molecules was chosen to keep the same mass ratio between DOX and CNT as in the optimal adsorption case in the experiment for modified MWCNTs (m_DOX_/m_CNT_ = 4/1). The purpose of this approach was to check the possibility of achieving high adsorption in unmodified systems in more favorable, anhydrous conditions considering only non-bonded interactions.

## 4. Conclusions

To render the carbon nanotubes a more effective drug career and to understand and predict interactions and adsorption mechanisms, we have modified the surface of MWCNTs and investigated the adsorption of DOX. The data show that surface modification increased the adsorption capacity of MWCNTs at least 20 times. MD simulations have confirmed the poor tendency to DOX adsorption on hydrophobic pristine CNTs. Theoretical calculation exhibited a dominant tendency of the drug to aggregate and less effective adsorption on the carbon surface. For the oxidized MWCNTs, the kinetic studies confirmed a strong influence of chemisorption in the adsorption process and a contribution of intra-particle diffusion that controls the uptake of DOX on the modified MWCNTs. The results of MD simulation are in qualitative agreement with the experimental data. Moreover, the simulation permitted observation of DOX aggregation and on this basis, it was possible to conclude that this process runs along two pathways: DOX is adsorbed on the nanotube wall and simultaneously the free DOX molecules undergo self-aggregation.

## Figures and Tables

**Table 1:** Chemical composition determined via EDS.

Material	Carbon (wt %)	Nitrogen (wt %)	Oxygen (wt %)	Chlorine (wt %)
MWCNTs	97.84	1.22	0.94	0.00
modified MWCNTs	95.75	1.46	2.79	0.00
DOX-MWCNTs	74.28	3.36	17.42	3.52
DOX	64.10	3.85	25.84	6.21

**Table 2:** List of calculated isotherm parameters (± SD) for different models of DOX adsorption on pristine and modified MWCNTs at 298.15 K.

	Freundlich			Langmuir			Temkin		
	*1/n*	*k_F_*	R^2^	q_max_ mg g^−1^	*K_L_* mL g^−1^	R^2^	*b* kJ mol^−1^	*k_t_* mL g^−1^	R^2^
Pristine MWCNTs	0.1725 ± 0.009	64.99 ± 6.10	0.9918	185.2 ± 20.4	0.0492 ± 0.004	0.9985	0.104 ± 0.009	4.70 ± 0.04	0.9891
Modified MWCNTs	0.8234 ± 0.063	42.61 ± 8.53	0.9658	-	-	-	0.00165 ± 0.00053	0.0464 ± 0.009	0.8750

**Table 3:** List of parameters obtained from the kinetic models for the adsorption of DOX on modified MWCNTs.

Kinetic Model	Parameters	Values
Pseudo-first order	q_e_/mg g^−1^	2290 ± 173
	k_1_/min^−1^	0.0110 ± 0.0009
	R^2^	0.915
Pseudo-second order	q_e_/mg g^−1^	4029 ± 241
	k_2_/g mg^−1^ min^−1^	6.92 × 10^−5^ ± 0.16 × 10^−5^
	R^2^	0.999
Intra-particle diffusion	k_1_/mg g^−1^ min ^−1/2^	1058.6 ± 62.9
	C_1_/mg g^−1^	82.59 ± 2.4
	R^2^	0.991
	k_2_/mg g^−1^ min ^−1/2^	77.12 ± 1.7
	C_2_/mg g^−1^	317.0 ± 21.3
	R^2^	0.903
Fractional power	v	0.283 ± 0.09
	K_FP_	1264.4 ± 74.4
	R^2^	0.859
Elovich model	α/mg g^−1^ min ^−1^	4136.2 ± 167.7
	β/g mg^−1^	0.001537 ± 0.00001
	R^2^	0.904
